# Neuropsychiatric Sequelae of COVID-19: A Review of Acute and Long-Term Manifestations

**DOI:** 10.1192/j.eurpsy.2025.1267

**Published:** 2025-08-26

**Authors:** M. Demetriou, V. Anagnostopoulou, M. Peyioti, V. Markatis, P. Argitis

**Affiliations:** 1 Psychiatry Department, General Hospital of Corfu, Corfu; 2 University General Hospital of Alexandroupolis, Alexandroupolis, Greece

## Abstract

**Introduction:**

The SARS-CoV-2 virus, responsible for COVID-19, has been shown to affect the central nervous system (CNS), leading to a wide range of acute and long-term neuropsychiatric symptoms. These symptoms can profoundly impair cognitive and emotional functioning, highlighting the need for a deeper understanding of their underlying mechanisms and clinical manifestations. Addressing these sequelae is critical for developing effective management strategies and improving patient outcomes. Long COVID has been associated with conditions such as brain fog, depression, anxiety, and fatigue, which can further exacerbate pre-existing mental health conditions and impair quality of life.

**Objectives:**

This review aims to examine the neuropsychiatric manifestations associated with COVID-19, focusing on both acute and long-term (long COVID) effects, and to explore potential pathophysiological mechanisms, including neuroinflammation and immune dysregulation.

**Methods:**

A systematic review of literature was conducted by searching databases such as PubMed and Scopus, using terms like ‘COVID-19’, ‘neuropsychiatric symptoms’, ‘long COVID’, ‘inflammation’, and ‘CNS involvement’. Only peer-reviewed articles published between 2020 and 2023 were included, with an emphasis on studies reporting acute neuropsychiatric symptoms and post-acute sequelae of COVID-19.

**Keywords:**

COVID-19, neuropsychiatric symptoms, long COVID, neuroinflammation, interleukin-6, CNS involvement

**Results:**

Acute neuropsychiatric manifestations of COVID-19 include encephalopathy, delirium, seizures, and mood disturbances. Approximately 22.5% of COVID-19 patients present with neuropsychiatric symptoms during the acute phase, including anxiety, depression, and cognitive impairment. In long COVID, persistent symptoms such as fatigue, depression, anxiety, sleep disorders, and cognitive dysfunction have been reported, with neuroinflammation and elevated interleukin-6 (IL-6) levels proposed as key mechanisms. Neuropsychiatric symptoms are observed in both hospitalized and non-hospitalized individuals, with risk factors including severe infection, female sex, and pre-existing mental health conditions.

**Conclusions:**

COVID-19 is associated with a broad spectrum of neuropsychiatric symptoms that persist beyond the acute phase. The underlying pathophysiology likely involves immune dysregulation, cytokine-mediated neuroinflammation, and direct viral invasion of the CNS. Early recognition and targeted interventions are essential to mitigate long-term neuropsychiatric complications.

**Disclosure of Interest:**

None Declared

